# Effect of dietary selenium intake on gut microbiota in older population in Enshi region

**DOI:** 10.1186/s41021-021-00220-3

**Published:** 2021-12-13

**Authors:** Zi-xiong Zhang, Hua Xiang, Guo-gen Sun, Yan-hua Yang, Chen Chen, Tuo Li

**Affiliations:** 1 Institute of Selenium and Human Health of Hubei, Hubei Province Enshi City, China; 2 Central Hospital of Enshi Autonomous Prefecture, Enshi Autonomous Prefecture, Hubei Province Enshi City, China; 3 Research Institute of Otolaryngology Head and Neck Surgery, Renmin Hospital of Wuhan University, Wuhan, China

**Keywords:** Selenium, Gut microbiota, 16S RNA, Diet, Enshi

## Abstract

**Background:**

The microbial ecosystem in the human gut varies between individuals with differences in diet. Selenium is one of most common trace elements in everyday diet, and selenium intake affects the human gut microbiota. We studied the effect of selenium intake on the gut microbiota in regions of Enshi with different distributions of selenium.

**Methods:**

One hundred elderly subjects (>65 years) were recruited from high-selenium and low-selenium areas in Enshi and blood, nail, and fecal specimens were obtained. The selenium contents in these samples were determined in triplicate by hydride generation atomic fluorescence spectrometry. DNA was extracted from fecal specimens and the microbial diversity was analyzed by 16 S RNA.

**Results:**

The selenium contents in the blood and nails were significantly different between the high- and low-selenium areas, and the composition of the intestinal microbiota, including abundance and extent of intestinal flora, was altered. The function and metabolic pathways of the gut microbiota showed clear differences.

**Conclusions:**

As a trace element in human diet, selenium intake is an important factor that affects the intestinal microbiota and is likely involved in many human diseases. This study provides new clues and ideas for studying the correlation between selenium and human health.

## Introduction

Human intestines occupy a fairly large surface area ranging from 30 to 400 cubic meters, [1], and the gut harbors more than 10^14^ microbes [2]. Human gut microbial communities are composed of the dominant phyla Firmicutes and Bacteroidetes and very low proportions of Proteobacteria, Acinobacteria, Fusobacteria, and Verrucbacteria [3]. They consist of approximately 1150 prevalent species, most of which are shared in population, and at least 160 such species are present in each individual [4, 5]. The gut microbiota is regarded as a “superorganism” and vastly expands on the functional capabilities provided by our genomes. The gut microbiome is estimated to contain 150 times more genes than the human genome [6]. Numerous studies have revealed that intestinal microbiota affect many aspects of host physiology including nutrient absorption, vitamin production, metabolic phenotype [7], and immune system development and regulation [7, 8]. The dysbiosis of intestinal microbiota could lead to host disease such as diabetes type 1 and type 2 [9], irritable bowel syndrome [10], colonic cancer [11], and inflammatory bowel disease [12].

Recent studies have indicated that the microbial ecosystem in the human gut varies between individuals based on country of origin, lifestyle, age, and health status [6]. The microbial ecosystem mainly reflects certain functional core microbiomes, wherein wide arrays of microbial genes are shared and identified [13]. Moreover, the functional core microbiomes are associated with different host phenotypes including country of origin and gender, leading to the proposal of three predominant “enterotypes”: Bacteroides, Prevotella, and Ruminococcus [14]. The aging process significantly affects human gut microbiota communities because of age-related physiological changes such as digestive hormone secretion, intestinal morphology, nutrient absorption, and functionality of the host immune system [15]. Diet was hypothesized to have a more significant impact on the microbial ecosystem and plays a crucial role in shifting the intestinal microbiota. Food source could lead to changes in circular pheromones in the host by affecting symbiotic bacteria [16]. Diets with high caloric content, high fat content, or low fiber content may change alter composition of the intestinal microbiota [17].

Trace elements, which are important in the composition of a diet, can also affect the intestinal microbiota. For example, iron supplementation contributes to the re-establishment of the original gut microbiota composition [18]. Selenium is one of most common trace elements in the diet, but few studies have reported the effect of selenium on the intestinal microbiota. The effect of selenium intake on the gut microbiota require further investigation. Enshi has the only separate selenium mines in the world, which were formed during the Maokou, Late Permian period. Carbon-siliceous sediments, also known as “stone coal”, contain the highest content of selenium (up to 8,500 mg/kg) [19]. Human activities have played important roles in manifesting a special geographical environment with uneven distributions of Se. We herein studied the effect of selenium intake on the gut microbiota in areas with different distributions of selenium.

As a trace element, selenium plays a variety of important roles in organisms, of which the presence of selenoprotein in the form of selenium plays an important role. The physiological metabolic activities that selenoprotein participates in include the body’s peroxide damage repair, participation in the regulation of hormone activity, and lipid component metabolism. Selenium in selenium. The existing form of protein is selenocysteine, and selenoenase is the most important type of selenoprotein. Food is the main way for the body to take in selenium. Selenium is widely found in foods such as eggs, fish, grains, meat, fruits, vegetables, nuts, etc., and it exists in the form of inorganic selenite. selenate, they are absorbed by plants from the soil and converted into organic form, in the form of selenomethionine and selenocysteine, Humans can obtain selenium by ingesting plant or animal food. Therefore, to study the effects of selenium on the human body, the soil selenium content can be used as a reference basis [20].

## Materials and methods

### instrumentation

**Table:** 

Instrument Name	Model
DNA isolation kit	PowerMax (stool/soil) DNA isolation kit (MoBio Laboratories)
Atomic fluorescence spectrometry	Titan instruments AFS-921 (Beijing Titan)
Sample collecter	Beckman Coulter Hemoccult II SENSA® cards (Beckman Coulter, CA)
DNA extract and purification kit	QIAamp DNA Stool Mini Kit
Spectrophotometer	NanoDrop 1000 spectrophotometer (Thermo Scientific, USA)
DNA sequencer	Illumina Hiseq4000 sequencer

### Ethical considerations

This study was conducted in accordance with the guidelines of the Declaration of Helsinki, and all procedures involving human subjects were approved by the Ethics Committee of the Central Hospital of Enshi Autonomous Prefecture. All subjects were given oral and written information about the purpose and procedures of the study. Consent to participate was signed by the subjects before the study started, and the subjects were free to withdraw from the study at any time point without giving any explanation.

### Participants

Subjects were recruited from high-selenium and low-selenium areas of Enshi, Hubei province, China. In total, more than 260 elderly (>65years) male and female adult volunteers were invited for screening. From this pool of volunteers, 100 subjects were randomized to participate in the study. The inclusion criteria were as follow: (1) lived in this area for more than ten years; (2) ability and willingness to understand and comply with the study procedures and sign the written informed consent; (3) no special diet customs; (4) willingness to provide blood, nail, and fecal specimens; (5) no use of antibiotic, antiviral, or antifungal drugs within the nearest month; and (6) blood selenium patiancecontent is >120 µg/L in high-selenium area and <80 µg/L in low-selenium area. The exclusion criteria were as follows: (1) suffered from acute gastrointestinal disease recently; (2) subjected to acute gastrointestinal surgery recently; (3) residents of abnormal nutritional status, such as terminal cancer patients or patients with cerebral apoplexy; and (4) did not provide blood, nail, or fecal specimens.

### Laboratory analyses

The selenium contents in all samples were determined in triplicate by hydride generation atomic fluorescence spectrometry.

### Fecal sample collection

Fecal samples were collected from the subjects using the two sections of Beckman Coulter Hemoccult II SENSA® cards (Beckman Coulter, CA) at home. The volunteers were told to collect specimens in advance, and the specimen should come from the end of the stool while avoiding contact with urine or other possible pollutants. The collected samples were immediately placed in a prepared centrifuge tube, which were kept in ice and sent to our laboratory. The samples were placed into plastic bags and stored at –80 °C until processed further. DNA was extracted from the 0.18–0.2 g fecal samples in accordance with the instructions of the QIAamp DNA Stool Mini Kit (Qiagen, USA). The DNA concentration and quality were determined by a NanoDrop 1000 spectrophotometer (Thermo Scientific, USA).

### Measurement of gut microbiota

#### DNA extraction and rDNA sequencing

DNA was extracted from all samples using the PowerMax (stool/soil) DNA isolation kit (MoBio Laboratories). All paired samples were evaluated by 16S rDNA primer (amplicon) Sequencing (515F (5’-GTGCCAGCMGCCGCGGTAA-3’) and 806R (5’-GGACTACHVGGGTWTCTAAT-3’)). Libraries of 16 S rDNA amplicons were prepared by targeting the V4 hypervariable regions of the rDNA. The amplicons were attached with adapters, and indexes were added in a subsequent nested polymerase chain reaction (98 °C for 30 s, 8 × (98 °C for 10 s, 55 °C for 20 s, 72 °C for 20 s), 72 °C for 5 min). Products from polymerase chain reaction were pooled and the purified with magnetic beads and sequenced on the Illumina Hiseq4000 sequencer (2 × 150 bp paired end).

#### Reads processing and metagenomic clustering

We used QIIME v1.9.0 to process reads and trim additional quality, demultiplexing [21]. Operational taxonomic unit (OTU) was picked using Vsearch v1.11.1, dereplicating, clustering, detection of chimeras [22]. Taxonomic assignment of individual datasets was performed using SILVA128. Alpha diversity was performed with qiime, including index of observed species. Beta diversity was calculated using QIIME with the matrix of (weighted and unweighted) Unifrac distance. Functional profiling of microbial communities was created with PICRUSt1.1.0 to predict the using 16 S rRNA sequences. Further analysis was processed with Statistical Analysis of Metagenomic Profiles (STAMP) software package v2.1.3 [23]. The beta diversity on taxonomy and functions based on Meta-Storms distances were generated by Parallel-META 3 (version 3.3.2) and phenotype-based functions were predicted with BugBase and FAPROTAX [24].

### Statistical analyses

Differences between two groups were determined by one-way analysis of variance. *p*-values of less than 0.05 were considered statistically significant. All statistical tests were carried out using SPSS version 17.0.

## Results

### Effect of selenium intake in diet on selenium content of blood and nails

According to the inclusion and exclusion criteria, 50 volunteers from three high-selenium areas and 50 from three low-selenium areas were randomized to participate in the study. The selenium content of their blood and nails were analyzed, showing clear differences (Table 1).

**Table 1:** Comparison of selenium content in the blood and nails between high- and low-selenium areas

Environment type	Region	Cases	Blood selenium concentration (µg/mL)	Nail selenium concentration (µg/g)
**High-selenium area**	**1**	**13**	**0.129 ± 0.900**	**0.837 ± 0.573**
	**2**	**20**		
	**3**	**17**		
**Low-selenium area**	**1**	**24**	**0.057 ± 0.165**	**0.362 ± 0.199**
	**2**	**26**		

### Degree of dietary selenium intake altered the intestinal microbiota composition

**Fig. 1:**
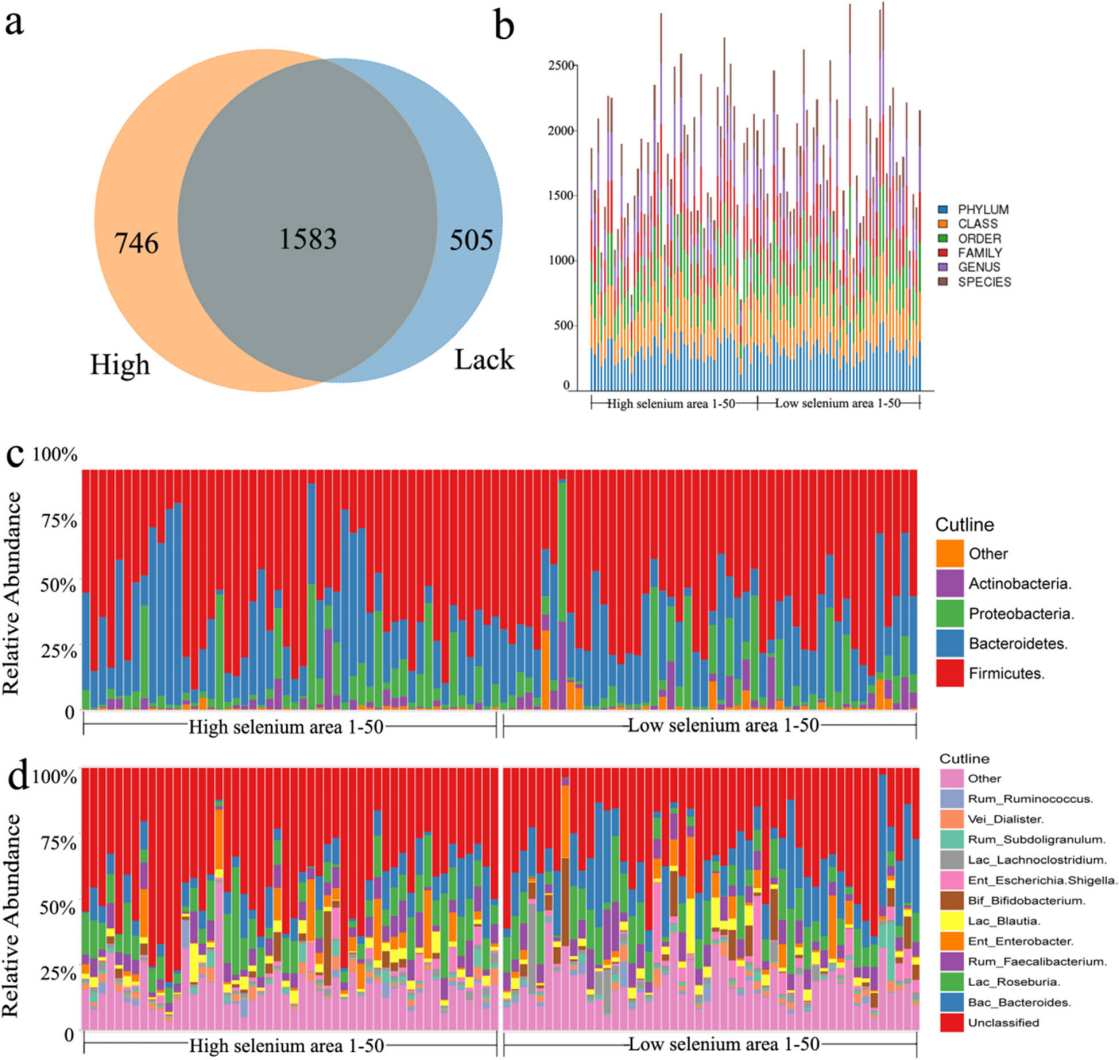
Difference in dietary selenium intake altered the composition of the intestinal microbiota. Fecal samples were collected from the high- and low-selenium groups and the intestinal microbiota were examined by 16 S rRNA sequencing. (**A**) Venn diagram of OTUs showing microbiota differences high- and low-selenium groups, with altered strains altered and overlap. (**B**) Microbial communities were examined based on phylum, class, order, family, genus, and species in the high- and low-selenium areas. (**C**) Relative abundance of predominant bacteria at the phylum level. (**D**) Relative abundance of predominant bacteria at the genus level

To compare fecal microbial populations of people living in high- and low-selenium area, the 16 S rRNA phylogenetic approach was applied. We observed 131,389 (84,066–145,017) effective tags in the sequencing. Sequences with at least 97 % similarity were clustered into OTUs and an average of 449 (236–744) OTUs were observed. A Venn diagram (Fig. 1A) displaying the overlapping OTUs for the two groups shows that 1583 of 2834 OTUs accounting for total richness were universal to all the samples, and 746 and 505 OTUs were observed in the high- and low-selenium groups, respectively. The microbial communities were assessed based on phylum, class, order, family, genus, and species between the high- and low-selenium area. Clear diversity was observed, but there was no significant difference between the two control groups (Fig. 1B). At the phylum level, Firmicutes were predominant in the two groups, followed by Bacteroidetes and Proteobacteria, with only a very small proportion of the other phyla. However, the proportion of Bacteroidetes and Proteobacteria was significantly different between the high- and low-selenium area (Fig. 1C). At the phylum level, Bacteroides, Roseburia, Faecalibacterium, Blautia, and Bifidobacterium are observed usually, but no significant difference was shown in the two control groups (Fig. 1D).

### Heatmap analysis of gut microflora in high- and low-selenium areas

**Fig. 2:**
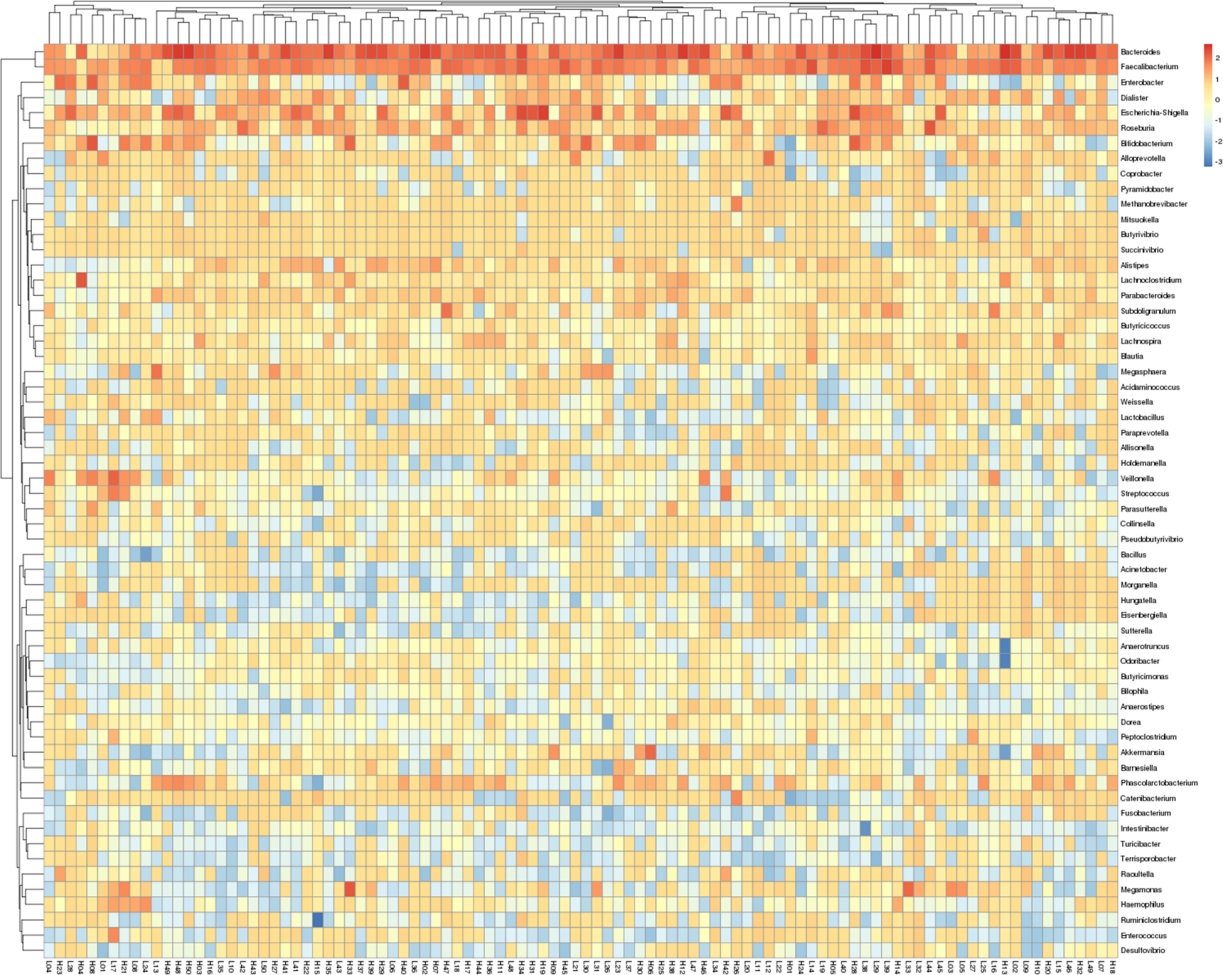
Heatmap of common genera in all samples from the high- and low-selenium areas. Lighter colors represents higher abundance. The degree of similarity between microbiota and between samples is indicated by the dendrogram on the x- and y-axis, respectively

Heatmaps were generated on the basis of the relative abundance of genera and produced by Bray–Curtis dissimilarity (Fig. 2). In total, the 60 most abundant genera of bacteria associated with the 100 volunteers are reflected by the heatmap. Of these, 10 genera were shared by most samples in a higher proportion, including *Bactericides, Faecalibacterium, Enterobacter, Dialister, Escherichia-shigella, Roseburia, Bilidobacterium, Alloprevotella, Coprobacter*, and *Pyramidobacter.* Further analysis showed that the relative abundance of genera has greater similarity within each group of volunteers.

### Beta diversity analysis of gut microflora in high- and low-selenium areas

**Fig. 3:**
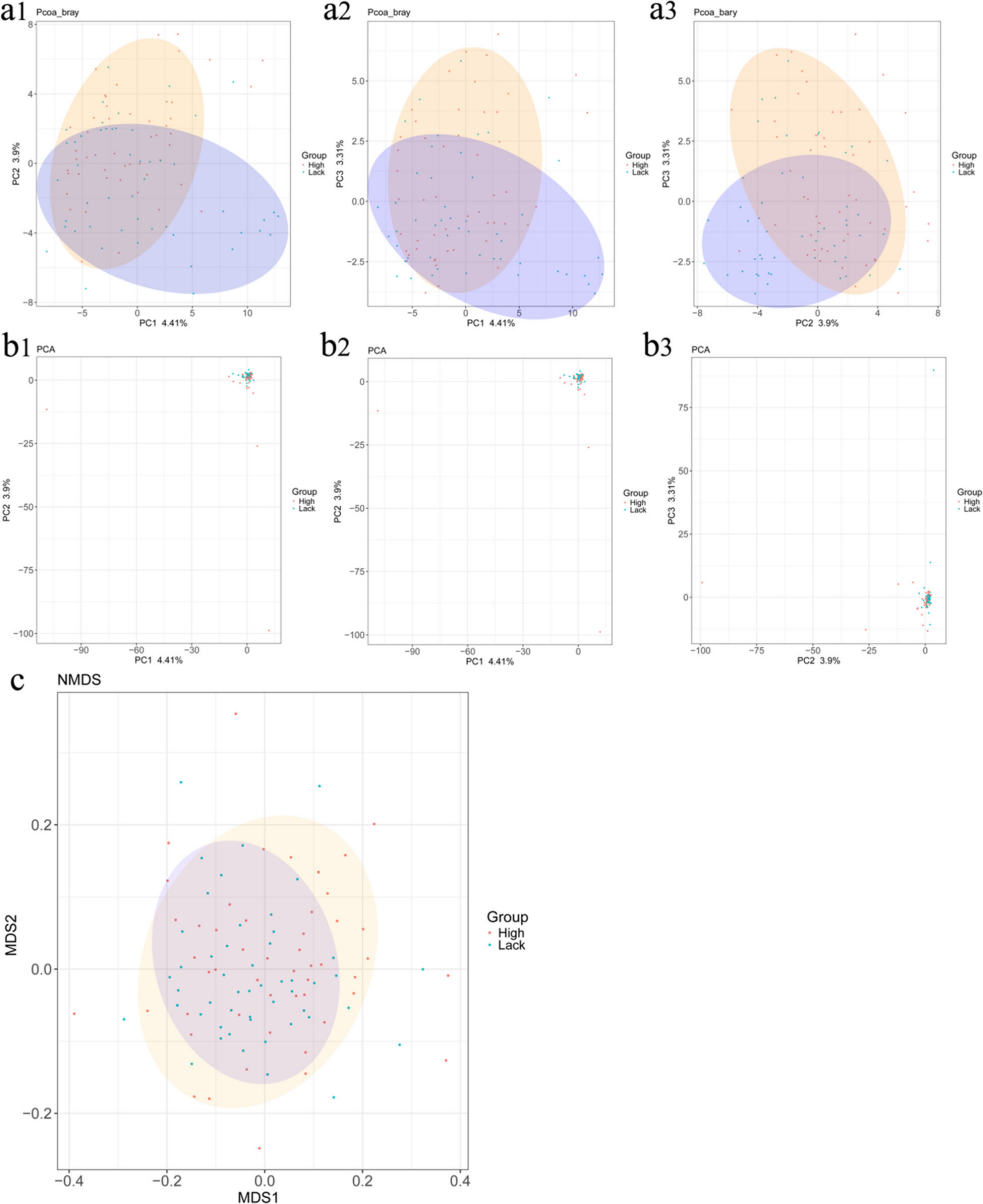
Beta diversity analysis of gut microflora in high- and low-selenium areas. Orange and blue represent high- and low-selenium area, respectively. **A1, A2, A3**, Principal co-ordinate analysis, a method of visualizing similarities or differences in data. Several features from the beta diversity of gut microflora were observed in three dimensions in high- and low-selenium areas. **B1, B2, B3**, Principal component analysis. Dominating features from the beta diversity of gut microflora were observed in three dimensions in high- and low-selenium areas. **(C)** Nonmetric multidimensional scaling comparing the difference between samples based on the number of distance matrices

Beta diversity analysis was performed to examine the differences between the samples as a method of visualizing similarities or differences in research data (Fig. 3). Each point represents a vector of top several main characteristic values. The red and blue points represent samples in the high- and low-selenium areas, respectively. Principal co-ordinates analysis showed a few features from three dimensions (Fig. 3A1, A2, A3). The orange and blue ellipses represent the main distribution of the high- and low-selenium areas, respectively, and the two groups showed significant differences. Principal component analysis showed dominating features from three dimensions (Fig. 3B1, B2, B3), with slight difference between the two groups. Nonmetric multidimensional scaling compares the differences between the two groups based on the number of distance matrices (Fig. 3B). Data from the high-selenium samples showed more discrete distribution compared to those from the low-selenium samples, with significant differences between them.

### PICRUSt, Clusters of Orthologous Groups (COG), and Kyoto Encyclopedia of Genes and Genomes (KEGG) analysis of gut microflora in high- and low-selenium areas

**Fig. 4:**
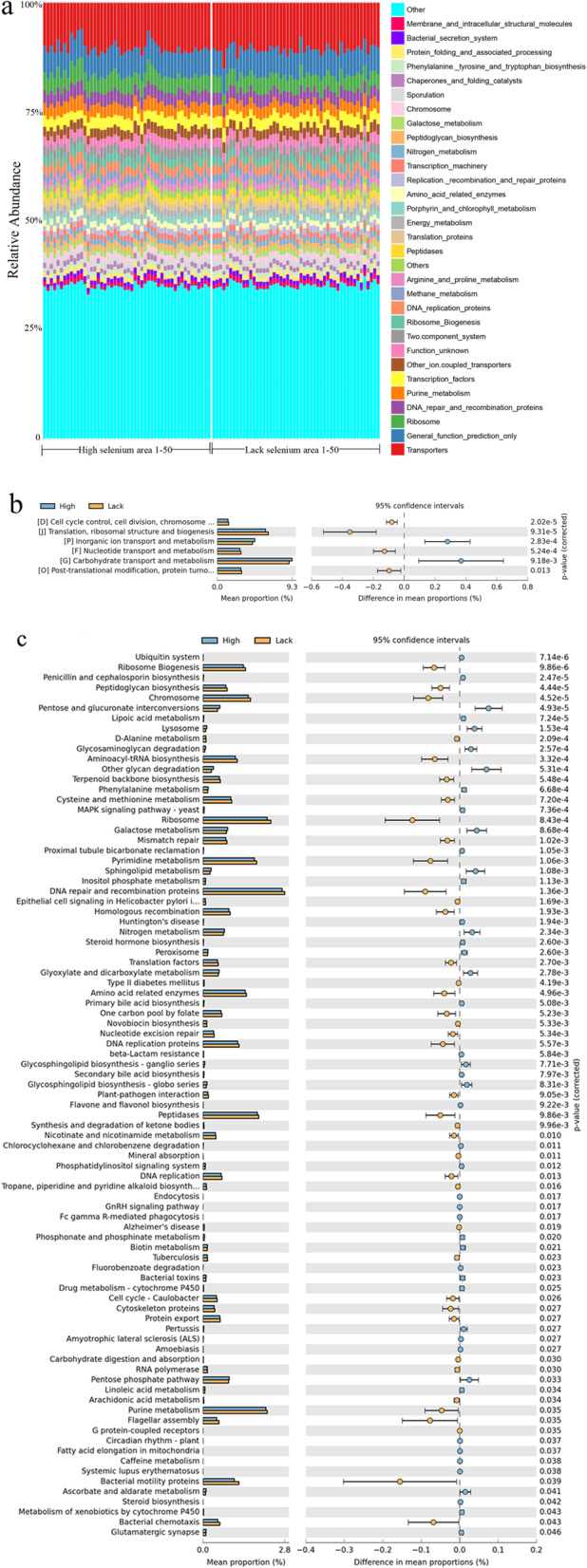
Variance analysis of gut microflora function in the high- and low-selenium areas. (**A**) Histogram of top 30 metabolic pathways analyzed by PICRUSt function prediction. Comparison of gut microflora function among 100 volunteers in high- and low-selenium areas. The histogram indicated slight differences in predicted function among individuals, but no significant difference was observed between the two groups. (**B**) COG analysis diagram indicating significant difference in cell cycle control, inorganic ion transport and metabolism, nucleotide transport and metabolism, carbohydrate transport and metabolism, post-translation modification, and ribosomal structure and biogenesis. (**C**) Variance analysis of gut microflora function by KEGG pathways in the third layer. Blue and orange represents high- and low-selenium, respectively. Diagram on the left lists the composition with significant differences between groups and proportion of each group, evaluated by KEGG in the third level. Diagram on the right indicates the confidence interval and p-value of each group

Output data from QIIME were analyzed using PICRUSt enabling the metagenomic make-up of samples to be inferred from the 16 S data. The top 30 metabolic pathways with significant differences were identified by PICRUSt function prediction. Gut microflora function was compared between 100 volunteers in the high- and low-selenium areas. The histogram indicated a significant difference in predicted function among individuals, but no significant difference between two groups (Fig. 4A). The metabolic profile of bacterial community structure of the 100 volunteers was annotated using COG and KEGG databases. Assembled contigs were analyzed by assigning predicted functions to genes based on COG. In total, 6 classes with significant differences were identified by COG based on functional categories, namely cell cycle control, inorganic ion transport and metabolism, nucleotide transport and metabolism, carbohydrate transport and metabolism, post-translation modification, and ribosomal structure and biogenesis (Fig. 4B). Comparison of fecal samples from high- and low-selenium samples showed significantly different metabolic functions in 87 pathways, as assessed by the level third of KEGG (Fig. 4C), particularly in pathways associated with amino acid synthesis and metabolism, lipoic acid metabolism, ubiquitin system, cysteine and methionine metabolism, DNA repair and recombination proteins, and nitrogen metabolism, glyoxylate and dicarboxylate metabolism and RNA polymerase.

### Phenotype classification of gut microflora in high- and low-selenium areas

**Fig. 5:**
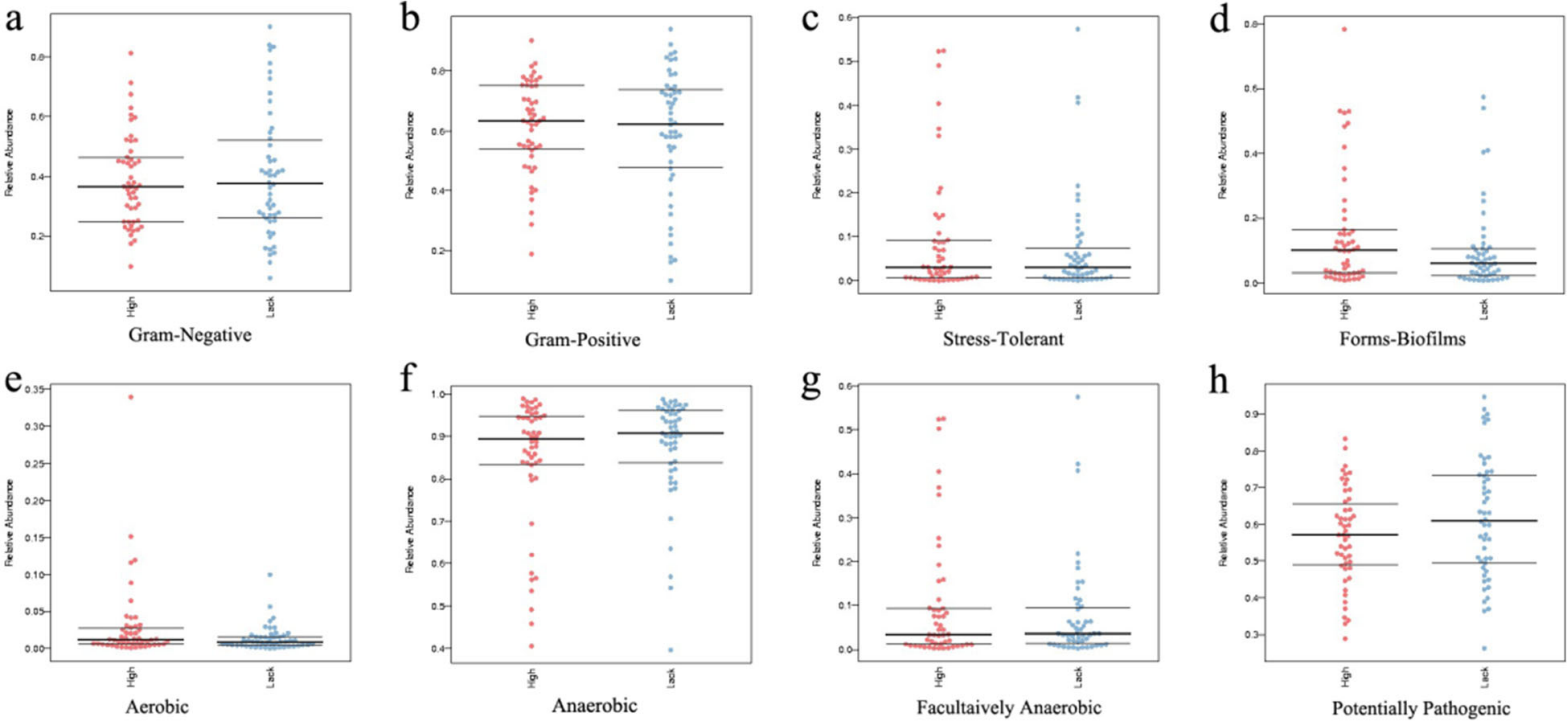
Comparison of eight phenotype classifications based on the result of 16 S high flux and based on BugBase. (**A**) Gram-positive phenotype. (**B**) Gram-negative phenotype. (**C**) Stress-tolerant phenotype. (**D**) Biofilm-forming phenotype. (**E**) Aerobic phenotype. (**F**) Anaerobic phenotype. (**G**) Facultatively anaerobic phenotype. (**H**). Potentially pathogenic phenotype

Comparison of eight phenotype classifications was carried out according to the results of the 16 s high flux and basing on BugBase. No significant difference was observed between the eight phenotype classifications from the high- and low-selenium areas, including gram-positive (Fig. 5A), gram-negative (Fig. 5B), stress-tolerant (Fig. 5C), biofilm-forming (Fig. 5D), aerobic (Fig. 5E), anaerobic (Fig. 5F), facultatively anaerobic (Fig. 5 G), and potentially pathogenic (Fig. 5 H) phenotypes.

## Discussion

Earlier studies have indicated that both high- and low-selenium areas exist, with significant variations in environmental selenium concentration [25]. The concentrations of selenium in the nails of the residents were strongly affected by the environmental selenium content, which was confirmed in this study (Table 1). The concentration of selenium in the blood and nail in the high-selenium area is significantly higher than that in the low-selenium area. In addition, residents in Enshi have similar genetic origins, living conditions, eating habits, and climate, providing a unique environment to study the correlation between selenium and human health. Thus, we focused on studying the relationship between selenium uptake and human gut microbiota base on this geographic feature.

In this study, there were several main reasons that elderly people were chosen as the subjects. In addition to obvious differences in dietary selenium intake, the living conditions of the subjects are relatively fixed, their life habits are similar, and they consume a basic diet. At the same time, there is a distinct association between age and gut microbiota composition [26]. The selenium content in the blood and nails confirmed that high- and low-selenium areas exhibited obvious differences. The intestinal microbiota is affected by many factors, such as environment, diet, and age. In this study, we showed that the dietary intake of the trace element selenium undoubtedly also has a significant impact on the intestinal microbiota. Our findings provide a valuable reference that can offer new clues and ideas for the study of selenium in human health.

We also observed distinct taxonomic associations of selenium in the gut microbiota, which were most apparent at the OTU level where a number of significant associations were observed between the two groups. Differences in dietary selenium intake altered the composition of the intestinal microbiota (Fig. 1). Significant differences in the number and distribution of OTUs were observed between the two groups, but it is also reflected in two aspects. One is the various bacteria distributed in phylum, class, order, family, genus, and species, and the other is the relative abundance of predominant bacteria at the phylum and genus levels. Heatmap analysis of common genera, beta diversity analysis, and phenotype classification based on BugBase showed a clear difference in gut microbiota between high- and low-selenium areas. Associating the above analyses with OTUs, the comprehensive results revealed that the difference in selenium intake is one of the main influencing factors that impact the intestinal flora. Further studies will be required to investigate the importance of specific taxa associated with selenium intake. This study has some limitations as the gut microbiota composition was only analyzed using 16 S rRNA amplicon analysis. The use of whole-metagenome sequencing would provide more accurate species level assignments and direct functional information [27].

This study focused on function prediction and metabolism pathways with PICRUSt, COG, and KEGG based on OTUs. Significant differences were identified in a number of metabolism pathways, indicating their association with selenium intake. Particularly, pathways involved in synthesis and degradation of ketone bodies, which were closely associated with cystic fibrosis [28], were observed. Metabolism pathways involved in type II diabetes were also observed, and its correlation with selenium has attracted the attention of medical researchers. Park et al. reported the correlation between higher toenail selenium content with lower risk of type II diabetes [29] and Stranges et al. studied the effects of long-term selenium supplementation on the incidence of type II diabetes [30]. It is reasonable to deduce that the gut microbiota plays an important role in the effect of selenium in type II diabetes. Metabolism pathways involved in Alzheimer’s disease (AD) is our focus because it is a current research highlight. Cardoso et al. [31] and Vural et al. [32] demonstrated that AD patients had lower selenium levels than healthy elderly people, and the effect of the gut microbiota on cognitive behavioral capability was demonstrated to play a critical role in the pathogenesis of AD through the microbiota-gut-brain axis [33]. It is clear that there is a close link between selenium, the gut microbiota, and AD, and this will be explored in future studies.

## Conclusion

In this study, we took advantage of the different distributions of selenium in the Enshi region and studied the effect of dietary selenium intake on the intestinal microflora. We showed that differences in the selenium intake in the elderly population significantly impacted the function and metabolism of gut microbes and may be associated with certain diseases. This study provides new ideas and directions for studying the correlation between selenium and human health.

## Data Availability

Not applicable.

## Abbreviations

OUT(s): Operational Taxonomic Unit(s)
PCoA: Principal co-ordinates analysis
PCA: Principal component analysis
NMDS: Nonmetric Multidimensional Scaling
AD: alzheimer’s disease
COG: Clusters of Orthologous Groups of proteins
KEGG: Kyoto Encyclopedia of Genes and Genomes
STAMP: Statistical Analysis of Metagenomic Profiles
